# Clinical and cognitive features associated with psychosis in Parkinson's disease: a longitudinal study

**DOI:** 10.3389/fnagi.2024.1463426

**Published:** 2024-11-06

**Authors:** Joseph L. Flanigan, Madaline B. Harrison, James T. Patrie, Binit B. Shah, Scott A. Sperling, Kathryn A. Wyman-Chick, William Alex Dalrymple, Matthew J. Barrett

**Affiliations:** ^1^Department of Neurology, University of Virginia, Charlottesville, VA, United States; ^2^Department of Public Health Sciences, University of Virginia, Charlottesville, VA, United States; ^3^Center for Neurological Restoration, Department of Neurology, Cleveland Clinic, Cleveland, OH, United States; ^4^HealthPartners Struthers Parkinson's Center, Saint Paul, MN, United States; ^5^Department of Neurology, Virginia Commonwealth University, Richmond, VA, United States

**Keywords:** Parkinson's disease, Parkinson's disease psychosis, minor hallucinations, cognitive function, depression

## Abstract

**Background:**

Parkinson's disease psychosis (PDPsy) is associated with increased nursing home placement and mortality and is closely linked with cognitive dysfunction.

**Objective:**

Assess the clinical and cognitive features associated with PDPsy in patients without dementia.

**Methods:**

We prospectively recruited people with Parkinson's disease (PwP) without dementia for a 3-year, longitudinal study at an outpatient movement disorders clinic. Participants completed annual visits involving assessment of motor and non-motor symptoms including neuropsychological testing. PDPsy was defined as the recurring presence of visual illusions, sense of presence, hallucinations, or delusions for at least 1 month. Using generalized estimating equations, we conducted two sets of analyses to separately assess the clinical and the cognitive predictors of PDPsy.

**Results:**

We enrolled 105 participants. At baseline, mean age was 67.8 (SD = 8.0), median disease duration was 4.9 years (IQR: 3.4–7.7), and mean MoCA was 24.8 (SD = 2.3). Prevalence of PDPsy increased over 3 years from 31% (*n* = 32) to 39% (*n* = 26). Forty-five participants (43%) experienced PDPsy. Visual illusions were most common (70%, *n* = 84), followed by hallucinations (58.3%, *n* = 70). In multivariate analysis, of the clinical variables, only depressive symptoms [OR 1.09, 95% CI: (1.03, 1.16), *p* = 0.004] increased the odds of PDPsy; of the cognitive variables, only Trail Making Test B-A scores [OR 1.43, 95% CI: (1.06, 1.93), *p* = 0.018] significantly increased the odds of PDPsy.

**Conclusions:**

In PwP without dementia, depressive symptoms were associated with increased risk of PDPsy. Executive/attentional dysfunction was also associated with PDPsy and may mark the transition from isolated minor hallucinations to more complex psychotic symptoms.

## Introduction

Psychosis is a common manifestation of Parkinson's disease (PD). Over a 12-year period, ~60% of patients with PD will experience hallucinations or delusions (Forsaa et al., 2010). Symptoms of psychosis occur along a spectrum from “minor” hallucinations (MH) to hallucinations, most often visual, and delusions. Visual hallucinations (VH) generally occur in later disease stages, worsen in severity over time, and may be accompanied by loss of insight (Ffytche et al., 2017). MH include visual illusions (misperceptions of real visual stimuli, for example, mistaking a coatrack for a person, or pareidolia, in which an object or face is perceived in formless objects, such as tree bark; Ffytche et al., 2017). MH also include “sense of presence” (a feeling that someone or some entity is present that cannot be attributed to real stimuli), and passage hallucinations (images briefly passing in peripheral vision; Ravina et al., 2007). MH may occur early in PD and may even precede motor symptoms (Pagonabarraga et al., 2016). Prior research has not clearly established the frequency with which MH progress to more complex psychotic symptoms (Lenka et al., 2019), such as well-formed VH, hallucinations in other modalities, and delusions.

PD psychosis (PDPsy) is associated with worse quality of life (Ffytche et al., 2017), greater nursing home placement (Ravina et al., 2007), increased mortality (Ravina et al., 2007), and greater caregiver distress (Ravina et al., 2007). Given these concerning outcomes, it is important to identify risk factors for PDPsy and to better understand the pathophysiology of PDPsy. Previously identified risk factors for symptoms of PDPsy include sex, age, duration and severity of PD, autonomic dysfunction, REM sleep behavior disorder (RBD), daytime somnolence, and cognitive impairment (Fénelon and Alves, 2010; Marinus et al., 2018). Cognitive impairment is one of the most frequent predictors of PDPsy (Fénelon and Alves, 2010), and previous research has sought to understand the close relationship between cognitive function and PDPsy.

Clinical factors associated with PDPsy have varied according to the criteria used to define it (Fenelon et al., 2010). Prior studies investigating clinical features associated with PDPsy generally concentrated on VH and/or delusions (Lenka et al., 2017). A National Institute of Health (NIH)-sponsored working group established comprehensive diagnostic criteria to define PDPsy which include delusions, VH, and MH (Ravina et al., 2007). Clinical correlates of PDPsy using these criteria have been less frequently investigated. Examining these associations can identify risk factors and inform understanding of the pathophysiology common to all PDPsy symptoms. Moreover, previous research on PDPsy has typically been cross-sectional (Lenka et al., 2017), highlighting a need for longitudinal studies to track the progression across the PDPsy spectrum and to better understand the associated clinical changes. To address these gaps, our study aimed to examine the phenomenology and clinical features of PDPsy over a 3-year period using the NIH criteria.

In addition, our study sought to evaluate the relationship between cognitive functioning and PDPsy. The neural systems involved in PDPsy are also implicated in cognitive processes, and theoretical models suggest cognitive impairment in certain domains is necessary to the development of PDPsy (Pagonabarraga et al., 2024). However, the cognitive architecture of PDPsy is not clearly understood. Better understanding this relationship could elucidate the mechanisms underlying PDPsy, inform etiological models, and facilitate novel treatments. In patients with PDPsy, past studies have observed deficits most often in executive function and attention, as well as in visual perception and memory (Lenka et al., 2017). However, most studies evaluated a single PDPsy symptom and there have been few longitudinal investigations. In order to better understand the cognitive profile of PDPsy, we utilized a multi-domain cognitive battery to assess which domains were associated with PDPsy over 3 years.

We conducted two sets of analyses: one assessing the clinical features associated with PDPsy and another to explore its cognitive profile. We hypothesized that cognitive impairment would be a key predictor of PDPsy in our clinical analysis, and that PDPsy would be characterized by executive dysfunction in our cognitive analysis.

## Materials and methods

### Participants

We recruited a convenience sample of patients without a diagnosis of dementia at the University of Virginia's outpatient Movement Disorders Clinic. Patients were identified by chart review or neurologist referral and met the following inclusion criteria: (1) ≥30 years old at onset of first motor symptom, (2) ≤ 85 years of age, and (3) diagnosis of idiopathic PD based on the UK Brain Bank criteria (i.e., presence of bradykinesia and at least one of three other cardinal PD features: resting tremor, rigidity, or postural instability). We excluded patients with a clinical diagnosis of dementia and/or a score on the Montreal Cognitive Assessment (MoCA) indicative of dementia (MoCA <21; Dalrymple-Alford et al., 2010). We additionally excluded patients with atypical parkinsonism and those who were participating in an experimental trial to treat PD. Neuropsychological and psychiatric assessments were performed at baseline and annually for 3 years. Participants were enrolled beginning in March 2013 and assessed in follow up through September 2019. The sample size was determined by the number of participants that could be recruited during the study period. The study was approved by the University of Virginia's Institutional Review Board and all participants provided written informed consent.

### Assessment of psychosis

At each visit, researchers assessed participants for symptoms of psychosis over the past month using the Scale for the Assessment of Positive Symptoms (SAPS). The SAPS does not assess illusions or sense of presence, so these symptoms were queried in a supplementary structured interview, in which researchers asked participants “In the last month have you experienced any illusions, for example mistaking a lamp in a corner for a person?” and “In the last month have you experienced a sense of presence, for example that there is a person standing in the room behind you?” Researchers applied NINDS-NIMH diagnostic criteria for PDPsy (Ravina et al., 2007). Based on these criteria, participants were classified as having PDPsy if (1) the participant reported at least one recurrent or continuous symptom for at least 1 month, (2) the symptom was not associated with a medication or condition other than PD (Ravina et al., 2007). Passage hallucinations are not considered in the NINDS-NIMH PDPsy criteria and were not assessed in this study.

### Clinical assessment

We obtained demographic information and details of PD onset and diagnosis from participants at baseline. Participants reported their current medications at each visit, and levodopa equivalent daily dose (LEDD; Tomlinson et al., 2010) was calculated for anti-parkinsonian medications. At each visit a movement disorders neurologist (MJB) administered the Movement Disorder Society Unified Parkinson's Disease Rating Scale (MDS-UPRDS). All medicated patients were assessed in the ON state. Participants additionally completed a series of questionnaires at each visit, including the MDS-UDPRS patient questionnaire, REM Sleep Behavior Disorder Screening Questionnaire (RBDSQ), Epworth Sleepiness Scale (ESS), Scales for Outcomes in Parkinson's—Autonomic (SCOPA-AUT), and the Beck Depression Inventory-II (BDI-II). Participants self-reported frequency of sleeping medication use on a scale from 1 (not at all) to 4 (more than 3x/week).

### Neuropsychological assessment

At each visit, a trained psychometrician or neuropsychologist administered a neuropsychological battery that included the Montreal Cognitive Assessment (MoCA), Controlled Oral Word Association (COWA), semantic fluency (Animals and Actions), the Matrix Reasoning subtest of the Wechsler Adult Intelligence Scale-Fourth Edition (WAIS-IV_MR_), Hopkins Verbal Learning Test-Revised (HVLT-R), Benton Judgment of Line Orientation (JLO), and Trail Making Test A & B (TMT_A_ & TMT_B_). Raw scores on TMT_A_ were subtracted from those of TMT_B_ to attain TMT_B − A_. Derived scores of the TMT such as TMT_B − A_ remove the effects of motor speed from the TMT_B_ (Cavaco et al., 2013), a potential confound to the measurement of executive functioning in PD given people with Parkinson's experience different motor symptoms. In addition, researchers have argued that the derived scores offer a better measure of executive functioning than the TMT raw scores (Cavaco et al., 2013). Derived scores have been shown to be sensitive to executive impairment, and shown better correlations with other measures of executive functioning (Hester et al., 2005). Normative data from Heaton et al. (2004) were used to obtain standardized scores for COWA, Animals, TMT_A_, TMT_B._ Normative data from Lamberty et al. (1994) were applied to TMT_B − A_. Raw scores were used for Actions and JLO. Standardized scores for HVLT-R and WAIS-IV_MR_ were obtained using their respective manuals (Wechsler, 2008; Brandt and Benedict, 1997).

### Statistical analyses

Statistical analyses were conducted with the objective of identifying clinical variables and cognitive variables which are associated with patients reporting PDPsy at a study visit. We selected clinical and cognitive predictor variables that have previously been reported in the literature as associated with symptoms of PDPsy. In univariate and multivariate fashions, the set of clinical variables and the set of cognitive variables were independently examined by way of univariate binomial generalized estimating equation regression (GEE) and by way of multiple binomial GEE regression. Binomial GEE regression was selected as the analytical approach because it is a well-established approach for analyzing within-subject clustered binary outcome data and is applicable to our multiple-visit data collection scheme.

Categorical data were summarized by frequencies and percentages. Continuous scale data were summarized by the mean and standard deviation, or the median and interquartile range (IQR), of the empirical distribution.

Univariate and multivariate binomial generalized estimating equation (GEE) regression models were constructed to assess unadjusted (univariate) and adjusted (multivariate) associations between the presence or absence of PDPsy at the longitudinal scheduled visit and the and non-time varying (e.g., sex) and time-varying (e.g., MoCA score) predictors of the presence or absence of PDPsy at the longitudinal visits. It should be noted that binomial GEE univariate and multivariate regression was utilized to assess associations instead of traditional univariate and multivariate binomial logistic regression, because of the repeated measures aspect of the PDPsy data collection. Unlike traditional binomial logistic regression, binomial GEE regression does not require that the binary outcome variable observations (i.e., PDPsy status) to be statistically conditionally independent.

For the univariate and multivariate GEE regression analyses, the outcome variable (Y) was a binary indicator variable that distinguished between the presence of PDPsy (Y = 1) or the absence PDPsy (Y = 0) at the visit. The predictors of PDPsy included assessment scores for symptoms previously reported in the literature as being associated with PDPsy (Fénelon and Alves, 2010). The clinical predictors included: sex, age, MoCA score, a binary indicator for presence of mild cognitive impairment (MoCA <26; Pagonabarraga et al., 2024), duration of disease from symptom onset, the presence of probable RBD (RBDSQ > 5; Nomura et al., 2011) MDS-UPDRS Part II score, MDS-UPDRS Part III score, ESS score, BDI-II score, SCOPA-AUT total score, LEDD, and a binary indicator for dopamine agonist use. Cognitive predictors included MoCA score (continuous), COWA, Animals, Actions, TMT_B_, TMT_A_, TMT_B − A_, HVLT-R Total Score, HVLT-R Delayed Recall, JLO, and WAIS-IV_MR_. No data were missing at any visit for age, sex, PD duration, MDS-UPDRS Part III score, LEDD, or the indicator for taking a dopamine agonist. Missing predictor data included 2 MoCA scores, 11 RBDSQ scores, 14 MDS-UPDRS Part II scores, 3 ESS scores, 7 BDI-II scores, and 4 SCOPA-AUT scores.

For the multivariate binomial GEE regression analyses, two multivariate GEE models were constructed. One to assess concomitant variable adjusted associations between the clinical predictor variables and PDPsy status at visits (Supplementary Equation 1) and the other to assess concomitant variable adjusted associations between the cognitive predictor variables and PDPsy status at visits (Supplementary Equation 1). For each multivariate binomial GEE regression model predictor variable, a Type III Wald chi-square test was used to test the null hypothesis that the predictor variable is not uniquely associated with the presence of PDPsy after concomitant variable (i.e., all remaining predictor variables) adjustment, vs. the alternative hypothesis that the predictor variable is uniquely associated the presence of PDPsy. A *p* < 0.05 decision rule was used as the null hypothesis rejection rule in both sets of analyses.

To assess for effects of participant attrition that might skew our sample, we compared baseline characteristics of participants who dropped out before the final visit and those completed it. Baseline categorical predictor variable comparisons were conducted by way of the Fisher Exact test (sex), and baseline continuous scaled predictor variable (age, MDS-UPDRS II and III, MoCA, and duration of disease) comparison were conducted with the Wilcoxon Rank Sum test.

## Results

### Patient sample

Of the 116 participants enrolled in the study, 11 participants with MoCA scores indicative of dementia were excluded. Of the remaining participants, 105 completed baseline visits, 92 completed year-1 visits, 74 completed year-2 visits, and 67 completed year-3 visits (see Supplementary Figure 1 for flow diagram of participation). The median length of participants' total follow-up time was 3.0 years [IQR: (1.9–3.0)]. The median time between annual evaluations was 12.2 months [IQR: (11.5–13.1)]. In the sensitivity analysis of baseline characteristics, only MoCA (*p* = 0.019) scores differed between participants who dropped out of the study early and those who completed the final visit (see Supplementary Table 1).

### Clinical characteristics

See Table 1 for participants' baseline clinical characteristics. Baseline characteristics of a slightly smaller sample of these patients (*n* = 101) are described in greater detail in a previous study (Barrett et al., 2017) Men accounted for 59% (*n* = 62) of participants. The mean age and standard deviation (SD) of participants was 67.8 ± 8.0 years, with a median disease duration of 4.9 years [IQR: (3.4–7.7)]. Three participants were not on anti-parkinsonian treatment at baseline, and two participants were taking anticholinergic therapy for treatment of Parkinson's at two visits during the study. Twelve participants reported taking a medication with anticholinergic properties for treatment of urinary symptoms for a total of 22 visits during the study. Two participants reported taking a cholinesterase inhibitor, both for two visits during the study. Six participants reported taking an antipsychotic medication for at least one visit during the study. Five participants were taking quetiapine and one participant did not report which antipsychotic medication they were taking. At each yearly visit the total number of participants taking an antipsychotic medication was three. Six participants had neurosurgical procedures to treat PD during the study. The median MDS-UPDRS part III score (motor score) was 24 [IQR: (16–36)]. The mean baseline MoCA score was 24.8 (SD = 2.3) indicating mildly impaired cognition.

**Table 1:** Participant baseline characteristics.

**Males, *n* (%)**	**62 (59.0)**
Education, y, μ (SD)	16.3 (3.0)
Age, y, μ (SD)	67.8 (8.0)
Age at PD diagnosis, y, μ (SD)	63.2 (8.9)
Duration of disease, y, median (IQR)	4.9 (3.4–7.7)
Total MDS-UPDRS Part III score, median (IQR)	24 (16–36)
LEDD Total, median (IQR)	525 (400–750)
Taking dopamine agonist, *n* (%)	31 (29.5)
MoCA score, μ (SD)	24.8 (2.3)

MDS-UPRDS, Movement Disorder Society Unified Parkinson's Disease Rating Scale; LEDD, Levodopa Equivalent Daily Dosage; MoCA, Montreal Cognitive Assessment; PD, Parkinson's Disease.

### Characteristics of psychosis

The point prevalence of PDPsy increased during the study from 31% (*n* = 32) at baseline to 39% (*n* = 26) at year three. The yearly incidence of PDPsy was 9% (*n* = 8) at year one, 4% (*n* = 3) at year two, and 3% (*n* = 2) at year three. For the 45 participants who met PDPsy criteria at least once during the study, the average number of visits at which they reported psychosis was 2.7 (SD = 1.1), and the average number of visits completed was 3.6 (SD = 0.76). PDPsy remitted for one visit in 14 of these participants, and for more than one visit in three participants.

At baseline, 19% (*n* = 20) of participants reported hallucinations, of which VH were most common (12%, *n* = 13); see Table 2. Ten participants (9.5%) reported isolated MH at baseline. The percentage of participants who reported isolated MH increased at year 1 (19%, *n* = 17) and year 2 (22%, *n* = 16). In contrast, at year 3, participants who reported isolated MH accounted for just 6% (*n* = 4) of participants, while 29.9% (*n* = 20) of participants reported hallucinations. The most common hallucinations at year 3 were visual (14.9%, *n* = 10), and multi-domain hallucinations accounted for a larger percentage of participants (10.4%, *n* = 7) at year 3 than at each previous visit. Of the 120 psychotic events over 3 years, visual illusions were the most frequently observed symptom (70%, *n* = 84), followed by hallucinations (58.3%, *n* = 70). Isolated MH accounted for 39.2% (*n* = 47) of psychotic events.

**Table 2:** Symptoms of psychosis by visit.

	**Baseline (*n* = 105)**	**Year 1 (*n* = 92)**	**Year 2 (*n* = 74)**	**Year 3 (*n* = 67)**
PD psychosis^*^	32 (30.5)	32 (34.8)	30 (40.5)	26 (38.8)
Hallucinations^*^	20 (19.0)	15 (16.3)	14 (18.9)	20 (29.9)
Auditory^*^	8 (7.6)	6 (6.5)	4 (5.4)	7 (10.4)
Visual^*^	13 (12.4)	7 (7.6)	4 (5.4)	10 (14.9)
Olfactory^*^	4 (3.8)	3 (3.3)	6 (8.1)	7 (10.4)
Somatic^*^	2 (1.9)	4 (4.3)	3 (4.1)	3 (4.5)
Multi-domain^*^	7 (6.7)	4 (4.3)	3 (4.1)	7 (10.4)
Delusions^*^	5 (4.8)	2 (2.2)	0 (0.0)	3 (4.5)
Minor hallucinations^*^	24 (22.9)	25 (27.2)	25 (33.8)	16 (23.9)
Visual illusions^*^	23 (21.9)	23 (25.0)	24 (32.4)	14 (20.9)
Sense of presence^*^	5 (4.8)	5 (5.4)	5 (6.8)	7 (10.4)
Isolated minor hallucinations^*^	10 (9.5)	17 (18.5)	16 (21.6)	4 (6.0)

^*^n (%).

PD, Parkinson's Disease.

### Clinical predictors of psychosis

Results of univariate binomial GEE regression analyses are summarized in Supplementary Table 2. All variables were significantly associated with psychosis in univariate analysis except age, MoCA score (continuous), and the indicator for presence of mild cognitive impairment (MoCA <26). We repeated the same set of univariate analyses using only the visits for which participants had complete data for all predictor variables; this had no impact on the results (data not shown). Using only the visits for which participants had complete data, BDI-II score was the only clinical variable to reach statistical significance in binomial GEE multivariate regression analysis (see Table 3). A higher BDI-II score increased the odds of a participant reporting symptoms of psychosis at a visit [OR 1.09, 95% CI: (1.03, 1.16), *p* = 0.004]. Box plots of BDI-II scores by year and presence/absence of PDPsy can be found in Supplementary Figure 2A.

**Table 3:** Multivariate adjusted odds ratios of clinical variables for reporting psychosis at a visit.

	**Ratio**	**Odds ratio**	**95% CI**	***P*-value**
Sex	Male: Female	1.63	0.65–4.04	0.294
Age	X + 1: X years	0.99	0.94–1.06	0.839
MoCA score	X + 1: X	1.02	0.86–1.20	0.828
PD-MCI (MoCA <26)	No: Yes	1.17	0.54–2.56	0.689
Duration of disease	X + 1: X years	1.06	0.96–1.18	0.271
RBDSQ score >5	>5: ≤ 5	1.48	0.73–3.01	0.275
MDS-UPDRS Part II score	X + 1: X	1.03	0.96–1.11	0.400
MDS-UPDRS Part III score	X + 1: X	1.01	0.98–1.04	0.526
ESS score	X + 1: X	0.99	0.91–1.08	0.884
BDI-II score	X + 1: X	1.09	1.03–1.16	**0.004**
SCOPA AUT score	X + 1: X	1.05	0.99–1.11	0.080
Taking dopamine agonist	Yes: No	2.43	0.99–5.88	0.054
LEDD	X + 100: X	0.96	0.85–1.08	0.529

Number of observations = 307.

CI, Confidence interval; MoCA, Montreal Cognitive Assessment; PD-MCI, Parkinson's Disease-Mild Cognitive Impairment; RBDSQ, REM Sleep Behavior Disorder Screening Questionnaire; MDS-UPRDS, Movement Disorder Society Unified Parkinson's Disease Rating Scale; ESS, Epworth Sleepiness Scale; BDI-II, Beck Depression Inventory-II; SCOPA-AUT, Scales for Outcomes in Parkinson's—Autonomic; LEDD, Levodopa Equivalent Daily Dosage. Bolded *p*-values indicate a statistically significant finding.

### Cognitive predictors of psychosis

Results of univariate binomial GEE regression analyses are summarized in Supplementary Table 3. COWA, Animals, Actions, TMT_A_, TMT_B_, and TMT_B − A_ scores were significantly associated with psychosis in univariate analyses. We repeated the same set of univariate analyses using only the visits for which participants had complete data for all predictor variables; this had no impact on the results (data not shown). Using only the 326 visits for which participants had complete data, only TMT_B − A_ score was significantly associated with psychosis in binomial GEE multivariate regression analysis (see Table 4). Worse performance on TMT_B − A_ increased the odds of a participant reporting psychosis at a visit [OR 1.43, 95% CI: (1.06, 1.93), *p* = 0.018]. Box plots of TMT_B − A_ *z*-scores by year and presence/absence of PDPsy can be found in Supplementary Figure 2B.

**Table 4:** Multivariate adjusted odds ratios of cognitive variables for reporting psychosis at a visit.

	**Ratio**	**Odds ratio**	**95% CI**	***P*-value**
MoCA, raw score	X + 1: X	1.09	0.97–1.22	0.132
COWA, *T*-score	X + 1: X	0.98	0.94–1.01	0.150
Semantic fluency, animals, *T*-score	X + 1: X	0.98	0.95–1.02	0.310
Semantic fluency, actions, raw score	X + 1: X	0.99	0.91–1.07	0.753
TMT_B_, *T*-score	X + 1: X	1.01	0.96–1.07	0.696
TMT_A_, *T*-score	X + 1: X	0.99	0.95–1.02	0.512
TMT_B − A_, *z*-score	X + 1: X	1.43	1.06–1.93	**0.018**
HVLT-R total, *T*-score	X + 1: X	1.02	0.97–1.08	0.350
HVLT-R delayed, *T*-score	X + 1: X	1.00	0.96–1.04	0.888
JLO, raw score	X + 1: X	0.96	0.89–1.05	0.376
WAIS-IV_MR_, scaled score	X + 1: X	1.08	0.95–1.23	0.245

Number of observations = 326.

CI, Confidence interval; MoCA, Montreal Cognitive Assessment; COWA, Controlled Oral Word Association; TMT, Trail Making Test; HVLT-R, Hopkins Verbal Learning Test-Revised; JLO, Benton Judgment of Line Orientation; WAIS-IV_MR_, Matrix Reasoning subtest of the Wechsler Adult Intelligence Scale-Fourth Edition. Bolded *p*-values indicate a statistically significant finding.

## Discussion

Our study assessed clinical and cognitive predictors of psychosis in a cohort of PD participants who on average exhibited mild cognitive impairment (MoCA = 24.8± 2.3) at baseline. Over 3 years, prevalence of psychosis in our sample ranged from 31 to 41%, and prevalence of VH was 5–15%. Though past studies have typically reported higher point prevalence of VH (22–38%), direct comparison with our study is complicated by differences in methodology and sample characteristics (Fénelon and Alves, 2010). Our application of NINDS-NIMH criteria for PDPsy, which require symptoms to be recurrent for at least the past month, and our sample's shorter duration of PD symptoms (median = 4.9 years) likely account for our lower prevalence of VH. A community-based study with patient characteristics comparable to our own that also applied NINDS-NIMH criteria found 26% prevalence of psychosis and 6.8% prevalence of VH (Mack et al., 2012). The slightly lower prevalence reported in this study relative to our cohort may be owed to its community-based rather than clinic-based assessment (Fénelon and Alves, 2010).

The present study assessed clinical predictors previously reported to be associated with symptoms of PDPsy (Fénelon and Alves, 2010). In multivariate analysis we found that a score indicative of greater depressive symptoms was the only significant clinical predictor of psychotic events. Although some studies have found no association, a majority of studies have linked depression and PDPsy (Fénelon and Alves, 2010; Morgante et al., 2012; Gibson et al., 2013; Factor et al., 2017; Aarsland et al., 1999; Marsh et al., 2004) Depressive symptoms have been found to be associated with both MH and complex symptoms of psychosis (Gibson et al., 2013; Fénelon et al., 2000). The frequent association of psychosis and depression in PD suggests they may have a shared pathology. A largescale case-control study found depression may precede the onset of motor symptoms in PD, suggesting depression is related to early PD pathology (Gustafsson et al., 2015). MH may also occur early in PD and prior to initiating dopamine therapy (Pagonabarraga et al., 2016). Lewy body pathology in the serotonergic raphe nuclei occurs in the initial stages of PD (Braak et al., 2004) and may contribute to the early occurrence of both depression and MH. Serotonergic systems are implicated in PDPsy through the use of pimavanserin, a 5HT2A inverse agonist, to treat symptoms of PDPsy (Cummings et al., 2014). Selective serotonin reuptake inhibitors (SSRIs) are commonly used to treat depression, and some case series have shown they improve symptoms of psychosis (Sid-Otmane et al., 2020). Cholinergic dysfunction has also been implicated in both PDPsy and PD depression (Marsh et al., 2004). PD depression has been associated with greater cortical cholinergic denervation while controlling for cognitive functioning (Bohnen et al., 2007) and degeneration in the cholinergic basal forebrain has been associated with PDPsy (Barrett et al., 2018). The cholinergic system is additionally implicated in PDPsy through its association with cognitive dysfunction in PD, which is one of the most reliable predictors of PDPsy (Marsh et al., 2004).

Cognitive impairment has often been characterized as the main disease-related risk factor for symptoms of PDPsy (Fénelon, 2008). However, our univariate and multivariate analyses did not evidence an association with either the MoCA or PD-MCI. Previous studies have predominantly focused on particular PDPsy symptoms (VH or delusions) that typically manifest later in the disease course (Lenka et al., 2019) when more severe cognitive impairment is also more likely to occur. Given the substantial proportion of psychotic events that were isolated minor hallucinations (39.2%), our sample can be considered on average to have earlier PDPsy than what most past investigations have examined. Our findings indicate that global cognitive impairment is not associated with early PDPsy; rather, a relationship may only emerge when both conditions are more advanced. Instead, depressive symptomatology may serve as an earlier risk factor for PDPsy. Consistent with this, in the Parkinson Progressive Markers Initiative (PPMI) dataset, which follows newly-diagnosed, drug-naïve patients, depression scores were higher at baseline for participants who went on to develop PDPsy (Ffytche et al., 2017). Patients and caregivers should be attentive to the onset of depressive symptoms, and clinicians should consider routinely assessing for and treating depression. Given PDPsy and depression may share pathophysiology, along with evidence that SSRIs can alleviate psychotic symptoms, it is possible early intervention for depression may help mitigate the risk of developing PDPsy. Future research should further investigate the relationship between depression and early PDPsy, as it may have important treatment implications and aid in the development of novel interventions.

It is notable that, in univariate analysis, probable RBD showed a strong association with PDPsy, increasing the likelihood of reporting it by 2.48, but there was no association in multivariate analysis. RBD can present prior to the symptoms of PD (Lenka et al., 2016) and has been found to be predictive of both VH and MH (Lenka et al., 2019). The absence of a relationship in our study may be attributable to the considerable overlap in risk factors for VH and RBD (Lenka et al., 2016). RBD has previously been associated with longer disease duration, more severe disease, greater dopaminergic medication requirement, increased risk of cognitive impairment, more depression, more excessive daytime sleepiness, and greater autonomic symptoms (Chahine et al., 2017), all variables for which we adjusted in multivariate analysis. The effects of RBD may thus have been confounded by the risk factors shared with PDPsy, which speaks to how inextricable these two disorders may be. Though it is of some debate, it has been theorized that RBD and VH are on a continuum, in which “kindling” with dopaminergic treatment leads to sleep disruptions, followed by vivid dreams, and then hallucinations and/or delusions (Lenka et al., 2016). While our study does not diminish the close relationship between RBD and VH, it suggests depressive symptomatology is an important element in the onset and evolution of PDPsy and should be incorporated into etiological models.

The present study also assessed the relationship between specific cognitive domains and PDPsy. A measure of executive and attentional functioning, TMT_B − A_, was the only significant cognitive predictor of psychotic events in multivariate analysis. In univariate analyses, all of our tests of executive functioning were also associated with psychosis, which underscores the relationship between psychosis and executive dysfunction in the study population. TMT_B − A_ requires shifting between mental sets (Seeley et al., 2007), and likely places greater demands on attention and working memory than the other executive tasks used in this study. These greater attentional demands may have been decisive in the association of TMT_B − A_ with psychotic events in multivariate analysis.

The previous cross-sectional study in this cohort did not find any significant associations between psychosis and cognitive test scores at baseline (Barrett et al., 2017). The longitudinal association of psychosis with TMT_B − A_ in the present study is likely attributable to advancing PD pathology. A larger proportion of events involving VH and multi-domain hallucinations were observed over 3 years than were observed at baseline. The development of these symptoms suggests dysfunction in the cholinergic basal forebrain and more pervasive cortical pathology (Ffytche et al., 2017), increasing the likelihood that cognitive function (and, especially, attention, and executive functioning) is also impaired. Indeed, worsening in TMT_B − A_ over 3 years can be observed in Supplementary Figure 2B. Given our finding that TMT_B − A_ was longitudinally associated with psychosis, impairments in executive/attentional functioning may mark a transition from MH to more complex psychotic symptoms. Clinicians and caregivers should be vigilant to the onset of executive/attentional impairment as this may signal early risk of developing or worsening PDPsy. Cholinesterase inhibitors (ChEIs) have been investigated in PDPsy with dementia, for which rivastigmine has the strongest evidence, improving both visual and non-visual hallucinations, though the effect size in meta-analysis was found to be small (Pagonabarraga et al., 2024). Only two participants in our cohort reported ChEI use despite it exhibiting mild cognitive impairment on average. This suggests that ChEIs are under-utilized in PD, though this may be owed to their worsening tremor in some patients (Pagonabarraga et al., 2024). Clinicians should consider early treatment of executive/attentional impairment with ChEIs as it is possible they may prevent onset or worsening of PDPsy and have the additional benefit of improving cognitive functioning (Pagonabarraga et al., 2024). Further investigations into the effect of ChEIs on early PDPsy symptoms is warranted.

The association between executive dysfunction and psychosis in our cohort is consistent with a majority of past studies. Executive dysfunction is the most frequently reported cognitive deficit in PDPsy (see Lenka et al., 2017 for review). Executive dysfunction in PD likely results from degeneration of basal forebrain nuclei (Braak et al., 2005). Thalamo-cortical circuits that connect the basal ganglion with the dorsolateral prefrontal cortex (PFC) and anterior PFC have also been implicated in executive dysfunction (Paulwoods and Tröster, 2003). In PD patients with VH, imaging studies have shown greater degeneration in frontal areas, including the dorsolateral and anterior PFC (see Alzahrani and Venneri, 2015 for review). Additionally, PD patients with VH have increased Lewy body density in frontal cortical areas (Gallagher et al., 2011).

In contrast to many past studies, we did not find an association between psychotic events and impairment in memory, visuospatial function, or semantic fluency. Our findings may be owed to our cohort's early PDPsy. Previous studies have typically not observed a relationship between cognitive functioning and MH (Lenka et al., 2019). One study found no significant cognitive differences between PD patients with isolated MH and those without (Llebaria et al., 2010). Two studies demonstrated an association between cognitive functioning and MH in PD. One found cognitive impairment (MoCA = 22–26) was predictive of daytime MH, but not those that occur after being aroused from sleep during the night or the early morning (Omoto et al., 2021). The other study found PD patients in the PPMI experiencing MH in the initial 5 years after diagnosis were more likely to be categorized as PD-MCI at 5 years, but cognitive categories did not reach significance in multivariate analysis (Bejr-kasem et al., 2021). However, these patients showed greater loss of gray matter at baseline, and were more likely to report cognitive decline at 5 years. These latter findings suggest cognitive dysfunction may be occurring in PD patients with MH prior to our ability to reliably detect it with cognitive testing (Bejr-kasem et al., 2021).

Our findings lend support to the attentional network model of VH in PD, which suggests hallucinations result from dysfunction within and between attentional networks (Shine et al., 2014). In particular, decreased activity in the Dorsal Attention Network (DAN), which is involved in directing attention to exogenous stimuli and priming sensory information, results in use of other attentional networks ill-suited to process this information (Shine et al., 2014). One such network is the Default Mode Network (DMN), which is associated with self-directed attention and semantic and episodic memory (Shine et al., 2014). In the setting of DAN impairment, the model suggests one way in which hallucinatory phenomena may manifest is through “over-reliance” on the DMN, resulting in the ascription of extraneous semantic or episodic memory to sensory stimuli (Shine et al., 2014). Impairment on TMT_B − A_ has been shown to be associated with decreased functional connectivity (FC) in the DAN in healthy patients (Seeley et al., 2007), and PD patients with hallucinations have demonstrated decreased activation in the DAN relative to those without (Shine et al., 2015). Additionally, PD patients with depression have shown increased FC in the DMN relative to those without depression (Lou et al., 2015). Sample sizes in these studies were small, and conflicting findings of network abnormalities in PD depression limit interpretation (Wei et al., 2017). However, taken together with our findings regarding the TMT _B − A_ and BDI they may indicate that executive dysfunction and the attentional burden of depression facilitate symptoms of psychosis through dysregulation of attentional networks.

Strengths of our study include its longitudinal assessment of psychosis in a cohort of patients without dementia, and our assessment of multiple cognitive domains. Past studies assessing cognition in PDPsy were largely cross-sectional, and tended to assess individual cognitive domains (Lenka et al., 2017). Considering the prevalence of isolated MH in our sample and their infrequent association with cognition in past research, it is important we observed a relationship between PDPsy and cognitive dysfunction. One limitation of our study is that NINDS-NIMH diagnostic criteria do not include passage hallucinations and so were not assessed. An additional limitation is that, owing to a lack of adequate power, we assessed the clinical category of PDPsy rather than conducted a granular analysis of the predictors of each symptom type. Evidence suggests risk factors for psychosis differ by phenomenology of symptoms, which likely have different pathological substrates (Ffytche et al., 2017). However, different symptom types co-occur and remit, making individual assessment challenging. Relatedly, given type and severity of PDPsy symptoms can change over the course of Parkinson's, consideration should be given to our population's early disease and mild motor symptoms when applying this study's findings to other populations. Additionally, participants who dropped out of the study early had lower baseline MoCA scores compared to those who completed it. As a result, our sample was skewed toward participants with better cognitive functioning. Other considerations for generalization include our population's high level of education and clinic-based recruitment. An additional limitation of our study is that we only assessed for psychosis over the month prior to a visit, which likely resulted in some participants being categorized as not having PDPsy or in remission at a visit despite having experienced symptoms during the year prior. Consequently, our assessment of psychosis is to some degree dependent on frequency of psychosis. Finally, we cannot rule out the possibility that the association between cognitive dysfunction and psychosis was a function of depression, which is known to affect performance on cognitive testing.

In summary, in a cohort of PD patients without dementia, we observed that more depressive symptoms increased risk of psychosis. Among cognitive domains, TMT_B − A_, a test of executive/attentional dysfunction was associated with psychosis. Importantly, these risk factors were observed in a cohort in which a sizable proportion of psychotic events were isolated MH. Future studies seeking to evaluate cognitive dysfunction in early PDPsy should consider employing cognitive assessments that place demands on executive and attentional functioning. In clinical settings, clinicians should be vigilant in assessing symptoms of depression and indications of executive/attentional dysfunction in PD, as they may signal risk for developing or worsening of PD psychosis.

## Data Availability

The raw data supporting the conclusions of this article will be made available by the authors, without undue reservation.
