# Supplementary material for: Clinical and cognitive features associated with psychosis in Parkinson's disease: a longitudinal study
Source: Front Aging Neurosci. 2024 Nov 6;16:1463426. doi: 10.3389/fnagi.2024.1463426 (PMC11579864; doi:10.3389/fnagi.2024.1463426)
Supplement: Supplementary file 1 [file Data_Sheet_1.docx]

***Supplementary Data***

*Supplementary Table 1. Comparison of baseline characteristics of participants who completed the final visit and those who dropped out*

|  | **Completers**  **(n=67)** | **Dropouts (n=38)** | **p-value** |
| --- | --- | --- | --- |
| Male Sex n (%) | 38 (56.7) | 26 (68.4) | 0.543 |
| Age, mean (SD) | 67.4 (7.2) | 68.6 (9.2) | 0.332 |
| MDS-UPDRS III; mean (SD) | 25.3 (13.0) | 29.6 (14.3) | 0.158 |
| MDS-UPDRS II; mean (SD)* | 10.2 (6.9) | 11.1 (7.5) | 0.490 |
| MoCA; mean (SD) | 25.2 (2.2) | 24.1 (2.5) | **0.019** |
| Disease duration (years); mean (SD) | 6.2 (4.1) | 5.6 (3.2) | 0.785 |

*Completers n=66, Dropouts n=35

*Supplementary Table 2. Univariate odds ratios of clinical variables for reporting psychosis at a visit*

| Predictor | Observations | Ratio | Odds ratio | 95% CI | P-value |
| --- | --- | --- | --- | --- | --- |
| Sex | 338 | Male: Female | 1.97 | 1.23-3.16 | **0.005** |
| Age | 338 | X + 1 : X years | 1.00 | 0.97-1.03 | 0.967 |
| MoCA score | 336 | X + 1 : X | 0.94 | 0.87-1.02 | 0.156 |
| PD-MCI (MoCA < 26) | 336 | No: Yes | 0.72 | 0.46-1.12 | 0.144 |
| Duration of disease | 338 | X + 1 : X years | 1.08 | 1.02-1.14 | **0.009** |
| RBDSQ score >5 | 327 | >5 : ≤ 5 | 2.48 | 1.56-3.94 | **<0.001** |
| MDS-UPDRS Part II score | 324 | X + 1 : X | 1.11 | 1.08-1.15 | **<0.001** |
| MDS-UPDRS Part III score | 338 | X + 1 : X | 1.03 | 1.02-1.05 | **<0.001** |
| ESS score | 335 | X + 1: X | 1.10 | 1.05-1.16 | **<0.001** |
| BDI-II score | 331 | X + 1: X | 1.12 | 1.08-1.16 | **<0.001** |
| SCOPA AUT score | 334 | X + 1: X | 1.10 | 1.07-1.14 | **<0.001** |
| Taking dopamine agonist | 338 | Yes: No | 1.76 | 1.10-2.81 | **0.019** |
| LEDD | 338 | X + 100: X | 1.11 | 1.05-1.19 | **0.001** |

Abbreviations: Confidence interval (CI), Montreal Cognitive Assessment (MoCA), PD-MCI (Parkinson’s Disease-Mild Cognitive Impairment), REM Sleep Behavior Disorder Screening Questionnaire (RBDSQ), Movement Disorder Society Unified Parkinson Disease Rating Scale (MDS-UPRDS), Epworth Sleepiness Scale (ESS), Beck Depression Inventory-II (BDI-II), Scales for Outcomes in Parkinson's - Autonomic (SCOPA- AUT), Levodopa Equivalent Daily Dosage (LEDD)

*Supplementary Table 3. Univariate odds ratios of cognitive variables for reporting psychosis at a visit*

| Predictor | Observations | Ratio | Odds Ratio | 95% CI | P-value |
| --- | --- | --- | --- | --- | --- |
| MoCA, raw score | 336 | X + 1 : X | 0.94 | 0.87-1.02 | 0.156 |
| COWA, T-score | 336 | X + 1 : X | 0.96 | 0.94-0.98 | **<0.001** |
| Semantic fluency, Animals, T-score | 336 | X + 1 : X | 0.96 | 0.94-0.98 | **0.001** |
| Semantic fluency, Actions, raw score | 335 | X + 1 : X | 0.93 | 0.89-0.97 | **0.002** |
| TMT_B_, T-score | 335 | X + 1 : X | 0.96 | 0.94-0.98 | **<0.001** |
| TMT_A_, T-score | 336 | X + 1 : X | 0.97 | 0.95-0.99 | **0.003** |
| TMT_B-A_, z-score | 335 | X + 1 : X | 1.35 | 1.18-1.55 | **<0.001** |
| HVLT-R Total, T-score | 336 | X + 1 : X | 0.99 | 0.97-1.02 | 0.563 |
| HVLT-R Delayed, T-score | 336 | X + 1 : X | 1.00 | 0.98-1.01 | 0.645 |
| JLO, raw score | 329 | X + 1 : X | 0.95 | 0.91-1.00 | 0.066 |
| WAIS-IV_MR_, scaled score | 331 | X + 1 : X | 0.96 | 0.88-1.04 | 0.317 |

Abbreviations: Confidence interval (CI), Montreal Cognitive Assessment (MoCA), Controlled Oral Word Association (COWA), Trail Making Test (TMT), Hopkins Verbal Learning Test-Revised (HVLT-R), Benton Judgment of Line Orientation (JLO), Matrix Reasoning subtest of the Wechsler Adult Intelligence Scale-Fourth Edition (WAIS-IV_MR_)

*Supplementary Figure 1. Flow diagram of participation.*


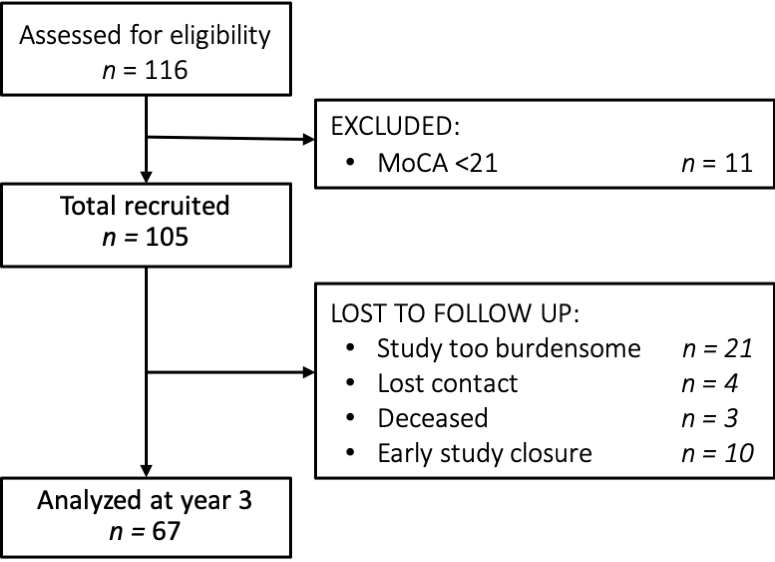


***Equation S1****. Generalized estimating binomial regression equation for predicting PD psychosis based on* ***clinical predictors***

**Eq S1**.  *logit(π) = -3.904 + 0.487 * I(Sex=Male) - 0.006 * Age (years) + 0.020 * MoCA score - 0.154 *I( PD-MCI [MoCA score < 26]) +0.058 * Disease Duration (years) + 0.395 * RBDSQ score + 0.031 * MDS-UPDRS Part II total score + 0.010 * MDS-UPDRS Part III total score - 0.006 * ESS score + 0.086 * BDI2 score + 0.048 * SCOPA-AUT total score + 0.890 * I(Taking Dopamine Agonist) + 0.0004* Levodopa Equivalent Daily Dose.*

Where π is the probability for reporting PD psychosis and logit(π) is the logit of π which equals log_e_ (π/(1-π)). I (⋅) is an indicator function, which is equal to 1 when the statement inside of the paraphrase is true and 0 otherwise.

***Equation S2****. Generalized estimating binomial regression equation for predicting PD psychosis based on* ***cognitive predictors****.*

**Eq S2.** *logit(π) = -1.696 + 0.088 * MoCA score + -0.025 * COWA T- score -0.017 * Animals T-score + 0.013 * Action raw score + 0.011 * Trails B score - 0.012 * Trails A score + 0.358 * Trails B score – Trails A z-score + 0.024 * HVLT total T-score – 0.003 * HVLT delayed T-score - 0.038 * JLO score + 0.077 * WAIS-IV_MR_ scaled score.*

Where π is the probability for reporting PD psychosis and logit(π) is the logit of π which equals log_e_ (π/(1-π)).

Supplementary Figure 2. (A) Empirical BDI-II score distributions stratified by visit and if PDPsy was reported at visit. Higher scores indicate worse depression. (B) Empirical TMT_B-A_ z-score distributions stratified by visit and if PDPsy reported at visit. Higher scores indicate more impairment. Interior box horizontal lines identify the median of the distribution, extent of box identifies the interquartile range, and lower and upper whiskers extend from the minimum to the maximum value of the empirical distribution.
